# MicroRNA-Based Triage of HPV-Positive Women Using Liquid-Based Cytology: Diagnostic Performance and Network-Level Insights

**DOI:** 10.3390/cancers18040559

**Published:** 2026-02-09

**Authors:** Justyna Pisarska, Aleksandra Kożańska, Rafał Rzepka, Katarzyna Baldy-Chudzik

**Affiliations:** 1 Department of Microbiology and Molecular Biology, Institute of Health Sciences, Collegium Medicum, University of Zielona Góra, 65-046 Zielona Góra, Poland; 2 Department of Gynecology and Obstetrics, Institute of Medical Sciences, Collegium Medicum, University of Zielona Góra, 65-046 Zielona Góra, Poland

**Keywords:** HPV infection, cervical intraepithelial neoplasia (CIN) lesions, cervical cancer, microRNA, biomarker, qPCR, miRNAs expression, receiver operating characteristic (ROC), Spearman correlation, regulatory network of miRNA

## Abstract

**Background:** Human papillomavirus (HPV)-based screening has substantially improved sensitivity for cervical cancer detection but remains limited by low specificity, leading to unnecessary colposcopy referrals. MicroRNAs (miRNAs) represent promising biomarkers for improving triage of HPV-positive women. This study evaluated the diagnostic and regulatory roles of selected miRNAs in cervical lesion progression using liquid-based cytology (LBC) specimens. **Methods:** Expression of six biologically relevant miRNAs (miR-15a-5p, miR-16-5p, miR-20b-5p, miR-155-5p, miR-34a-5p, and miR-140-3p) was analyzed across NILM, CIN II, CIN III, and cervical cancer (CC) samples. All miRNA analyses were performed using residual cellular material derived from the same liquid-based cytology (LBC) specimens collected during the HPV screening visit, without requiring any additional sampling prior to colposcopy. Diagnostic performance was assessed using ROC analysis. To capture regulatory dynamics beyond expression magnitude, correlation, and differential correlation (Δρ), network analyses were applied. **Results:** Stage-dependent changes in miRNA expression were observed across disease categories; however, expression magnitude alone did not fully explain diagnostic performance. Upregulated miRNAs, particularly miR-16-5p, miR-20b-5p, and miR-155-5p, demonstrated high diagnostic accuracy for distinguishing NILM from high-grade lesions and invasive cancer. In contrast, downregulated miRNAs showed limited diagnostic utility. Correlation analyses revealed progressive remodeling of miRNA co-expression networks, with the most pronounced changes occurring during the CIN II–to–CIN III transition. Notably, miRNAs with strong diagnostic performance did not uniformly function as network hubs, indicating distinct roles as biomarkers versus regulators of network dynamics. **Conclusions:** Cervical lesion progression is characterized not only by changes in miRNA expression levels but also by stage-specific reorganization of miRNA regulatory networks. Integrating diagnostic performance with network-level analysis enables improved identification of clinically robust triage markers and provides additional insight into regulatory instability associated with progression.

## Simple Summary

Current cervical cancer screening utilizes Pap smears and HPV DNA tests, which have high sensitivity but low specificity, as many of these infections are transient and leave no lesions in the cervix. This diagnostic approach has increased the number of tests and associated costs. Therefore, screening methods still require refinement to achieve greater diagnostic accuracy. In this study, we examined the utility of selected small, non-coding regulatory RNAs (miRNAs) as biomarkers to enhance the diagnostic accuracy of screening tests. Liquid-based cytology samples were used as the starting material for the analyses. We found that some miRNAs accurately distinguished women with advanced precancerous lesions or cancer from healthy women, while others reflected broader molecular instability associated with disease progression. Importantly, changes in miRNA interactions provided additional information beyond simple expression levels. These findings suggest that combining miRNA-based diagnostics with analysis at the miRNA regulatory network level may improve diagnosis and enable more precise disease risk stratification in screening.

## 1. Introduction

Cervical cancer is one of the most common malignancies of the female reproductive system [1]. Many factors influence the development of this life-threatening disease, including socioeconomic status, age at first intercourse, alcohol consumption, smoking, genetic predisposition, immunosuppression, and the number of pregnancies and deliveries, particularly in young women. Persistent infection with high-risk HPV (HR-HPV) is a key risk factor for developing cervical cancer [2]. Extensive preventive measures, such as vaccination and screening, have significantly contributed to the decline in the incidence of this cancer [3]. However, the Pap test, commonly used to screen women, often lacks sufficient sensitivity to detect cervical intraepithelial neoplasia (CIN) accurately [4]. Modern HPV DNA tests, despite their high sensitivity, have low specificity because most HPV infections are transient and do not always lead to cervical lesions. Therefore, screening methods still require refinement to achieve greater diagnostic accuracy. In particular, there is a growing clinical need for molecular triage strategies that can better stratify risk among HPV-positive women and reduce unnecessary colposcopy referrals, while remaining compatible with routine screening workflows [5,6,7].

Persistent HPV infection can lead to the development of precancerous lesions, which are associated with disruption of intracellular signaling in the infected cell, affecting the cell cycle and apoptosis. The progression of precancerous lesions is associated with a steady increase in the expression of the viral oncogenes E6 and E7, which in turn promotes HPV integration into the host genome. DNA repair mechanisms and signaling pathways gradually become impaired in the host cell, leading to changes at the transcriptional and epigenetic levels [7,8,9,10]. The increasingly better understanding of this complex system of connections in carcinogenesis creates a real opportunity to identify factors that can serve as biomarkers to assess the progression from a precancerous state to cancer.

Significant advances in RNA sequencing (RNA-seq) methods have shown that messenger RNA (mRNA) constitutes a small fraction of the human transcriptome compared to non-coding RNAs (ncRNAs) [10]. Within ncRNAs, miRNAs constitute a key class of short, regulatory RNAs, 19–25 nucleotides in length. MiRNAs bind to the 3′-UTR (3′-uncoding region) of mRNAs, regulating gene expression by promoting mRNA degradation or inhibiting protein synthesis [9]. MiRNAs regulate nearly 60% of all protein-coding genes, earning them the title of “master modulators” of the genome [10,11,12].

Over the past two decades, the role of miRNAs in cervical cancer carcinogenesis has attracted increasing research attention. Currently, approximately 2500 human miRNAs are registered in the miRBase database, and some have been annotated as oncogenes or tumor suppressors. Accumulating evidence indicates that miRNAs can act as oncogenes or tumor suppressors, depending on their target mRNA, in various types of cancer, including cervical cancer [13,14,15]. Changes in miRNA expression in cervical cancer are associated with genetic variations, including deletions, amplifications, and point mutations, as well as epigenetic modifications, such as DNA methylation [16,17,18]. Studies have shown that miRNAs are secreted into body fluids, including serum and vaginal secretions, suggesting their potential as biomarkers for early-stage cervical cancer [19,20,21]. Additionally, some miRNA loci overlap with HPV integration sites, suggesting that HPV may influence the expression of specific miRNAs. MiRNAs such as miR-125b and miR-34a are associated with an increased risk of HPV genome integration, regulation of HPV-E6 protein expression, and contribution to cervical cancer development [22,23,24]. Aberrant miRNA expression in cervical cancer and its precursor lesions has been studied. Differential expression of various miRNAs has been found in cancerous tissues compared to normal tissues [20,23,25,26,27]. However, the results of these studies have not always been consistent and ambiguous regarding the role of miRNAs in cervical cancer carcinogenesis. These differences may be due to different material sources and analysis methods [28,29].

Importantly, miRNA-based biomarkers are particularly attractive because they may potentially be implemented as reflex molecular assays using residual liquid-based cytology material collected during routine HPV screening, without requiring additional sampling [24,26].

This study aims to determine whether the expression profiles of six selected miRNAs—miR-15a-5p, miR-16-5p, miR-20b-5p, and miR-155-5p (with increased expression) and miR-34a-5p and miR-140-3p (with decreased expression)—can serve as biomarkers of precancerous lesions (CIN II, CIN III) and cervical cancer, analyzed in material from Liquid Based Cytology (LBC).

## 2. Materials and Methods

### 2.1. Study Population and Sample Collection

Between January 2020 and November 2023, women attending a cervical cancer screening program at the University Hospital in Zielona Góra (western Poland) were prospectively recruited. Recruitment and cervical specimen collection were conducted within an ongoing ethically approved HPV-focused research framework (Resolution No. 10/75/2017, 22 May 2017). Exclusion criteria were prior cervical cancer or other malignancies, previous cervical treatment, pregnancy, and age < 30 or >65 years.

Cervical samples were collected using a Cervex-Brush (Rovers Medical Devices, Oss, The Netherlands) and preserved in SurePath medium (Becton, Dickinson and Company, Franklin Lakes, NJ, USA) as part of the routine liquid-based cytology (LBC) workflow for Pap testing and HPV genotyping. Importantly, the same LBC sample obtained during this single screening visit served as the starting material for miRNA analysis, ensuring that the proposed molecular triage approach can be integrated into standard clinical practice without requiring additional sampling or extra patient visits. For miRNA profiling, residual exfoliated cervical cells remaining in the SurePath LBC vials after completion of cytological evaluation and HPV testing were processed. Aliquots of the cellular suspension were transferred into TRI Reagent Solution (Merck KGaA, Darmstadt, Germany) to stabilize RNA prior to extraction. This procedure was applied uniformly across all diagnostic categories, enabling consistent comparison of miRNA expression profiles between groups. For the purposes of the present study, miRNA expression analyses were performed as an approved extension of the protocol after additional ethics clearance was granted in November 2022 (Resolution No. 20/2022, 9 November 2022).

Cytology was evaluated according to the Bethesda System 2014 (NILM, ASC-US, LSIL, HSIL, SCC, AGC) [30]. Samples with normal cytology and negative HPV results served as healthy controls. In women referred for colposcopy due to HPV positivity, histopathological verification was performed using targeted biopsies when acetowhite lesions were present and classified according to IFCPC criteria [31].

A total of 100 women were included and assigned to histopathology-based groups: healthy controls (HPV−, *n* = 20), NILM with HPV infection (*n* = 20), CIN II (*n* = 20), CIN III (*n* = 20), and cervical cancer (CC; *n* = 20). Additionally, tissue biopsies from 15 patients undergoing LEEP/LLETZ for suspected invasion or carcinoma were collected in RNAlater (Thermo Fisher Scientific, Waltham, MA, USA) and stored at −20 °C for comparative miRNA analysis. Clinical and pathological characteristics are summarized in Supplemental Table S1. A unified overview of specimen collection timing, storage conditions, and downstream processing steps is provided in Supplementary Figure S1.

### 2.2. DNA Extraction and HPV Genotyping

Liquid-Based Cytology (LBC) samples were stored in SurePath medium at +4 °C for up to five days before DNA isolation. Exfoliated cells were concentrated to a volume of 2 mL (4200× *g*, 10 min) and counted in a Bürker chamber (Brand GmbH + Co. KG, Wertheim, Germany) immediately before extraction. Genomic DNA was isolated using the GeneJET Genomic DNA Purification Kit (Thermo Fisher Scientific, Waltham, MA, USA)) according to the manufacturer’s protocol for mammalian cells (up to 5 × 10^6^ cells). DNA concentration and purity were assessed spectrophotometrically using a NanoPhotometer (Implen GmbH, Munich, Germany). Purified DNA was stored at −80 °C until further analysis. HPV genotypes were detected using the Roche Linear Array HPV Genotyping Test (Roche Molecular Systems, Pleasanton, CA, USA), a PCR-based assay that identifies 37 HPV genotypes, including all significant high- and low-risk types.

The assay includes four internal quality controls: an HPV-16 positive control, low and high β-globin controls to verify DNA adequacy and amplification performance, and a negative control to exclude contamination.

### 2.3. Selection of Candidate miRNAs

The selection of miRNAs was based on an integrative approach combining public database analysis and literature review. Initially, the GEO2R database (https://www.ncbi.nlm.nih.gov/geo/geo2r); accessed on 28 November 2025)was used to identify miRNAs differentially expressed during progression from cervical intraepithelial neoplasia to cervical cancer and subsequently cross-validated using the dbDEMC database (https://www.biosino.org/dbDEMC/index; accessed on 2 December 2025), which curates miRNAs with confirmed differential expression across human malignancies. In addition, a targeted literature review [32,33,34,35,36,37] was conducted to identify miRNAs implicated in cervical lesion progression and cervical cancer prognosis. MiRNAs were selected for further analysis based on consistent differential expression in public datasets and association with CIN–CC progression. The selected miRNAs were chosen to reflect different stages of cervical disease progression and to enable the evaluation of their potential utility as minimally invasive biomarkers for risk stratification and disease progression monitoring in HPV-positive women. Based on this strategy, the following miRNAs were selected for downstream analyses: miR-15a-5p, miR-16-5p, miR-20b-5p, miR-155-5p, miR-34a-5p, and miR-140-3p.

### 2.4. RNA Isolation, Reverse Transcription, and RT-qPCR

Total RNA was isolated using TRI Reagent Solution (Invitrogen™, Thermo Fisher Scientific, Waltham, MA, USA) according to the manufacturer’s instructions. RNA quantity and purity were evaluated spectrophotometrically (Implen NanoPhotometer N60), and only samples with A260/A280 ratios > 1.8 were included. Reverse transcription of miRNAs was performed using the miRNA 1st-Strand cDNA Synthesis Kit (Agilent Technologies, Santa Clara, CA, USA)), which includes a polyadenylation step followed by first-strand cDNA synthesis. Quantitative PCR was conducted using LightCycler^®^480 SYBR Green I Master (RocheDiagnostics GmbH, Mannheim, Germany) on a LightCycler^®^96 system(Roche Diagnostics GmbH, Mannheim, Germany) in a final reaction volume of 10 µL. Expression levels of miR-15a-5p, miR-16-5p, miR-20b-5p, miR-155-5p, miR-34a-5p, and miR-140-3p were quantified. miR-423 and RNU43 were used as internal reference genes [38]. Primer sequences are listed in Supplemental Table S2. Relative miRNA expression levels were calculated using the 2^−^ΔΔCt method, with normalization based on the geometric mean of the two reference genes [39].

### 2.5. Statistical Analysis

Statistical analyses were performed using IBM SPSS Statistics for Windows, version 20.0 (IBM Corp., Armonk, NY, USA). Normalized miRNA expression values were log_2_-transformed. Fold change (FC) was calculated as the ratio of the median expression in each diagnostic category to the median expression in the control group (Control). Group differences were evaluated using Welch’s *t*-test to account for unequal variances. Statistical significance was defined as: **** *p* < 1 × 10^−4^; *** *p* < 1 × 10^−3^; ** *p* < 1 × 10^−2^; * *p* < 0.05. Diagnostic performance was assessed using receiver operating characteristic (ROC) curve analysis. The area under the curve (AUC) and the Youden index were calculated to evaluate discriminatory ability. Optimal cut-off values were determined using the Youden index, and sensitivity, specificity, positive predictive value (PPV), and negative predictive value (NPV) were calculated based on observed prevalence within each comparison. Correlation and Network Analysis: for each diagnostic category (NILM, CIN II, CIN III, CC), pairwise miRNA associations were assessed using Spearman’s rank correlation coefficients. To evaluate changes in miRNA regulatory relationships during disease progression, differential correlations (Δρ) were calculated as: Δρ = ρ advanced stage−ρ preceding stage. Differential correlation networks were constructed using a threshold of |Δρ| ≥ 0.3. Network topology was characterized by node degree, and miRNAs were classified as network hubs if their degree fell within the upper quartile of the degree distribution; remaining miRNAs were classified as stable.

## 3. Results

### 3.1. Associations Between miRNA Expression and Cervical Disease Progression

**Figure 1:**
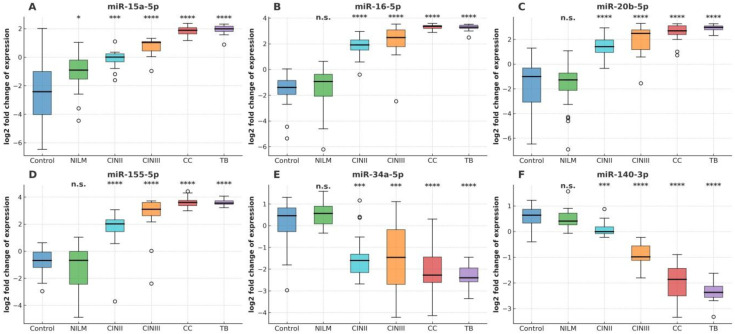
(**A–F**) Differential expression profiles of six miRNAs in exfoliated cervical cells from controls (Control, NILM), patients with precancerous lesions (CIN II, CIN III), cervical cancer (CC), and CC biopsy specimens (TB). (**A**–**D**) Up-regulated miRNAs: miR-15a-5p, miR-16-5p, miR-20b-5p, and miR-155-5p. (**E**,**F**) Down-regulated miRNAs: miR-34a-5p and miR-140-3p. Data are shown as boxplots of log_2_FC values relative to controls. Boxes represent the interquartile range, the central line indicates the median, and circles denote individual data points. Colors indicate different diagnostic groups. **** *p* < 1 × 10^−4^; *** *p* < 1 × 10^−3^; * *p* < 0.05; n.s., not significant.

This study evaluated the utility of selected miRNA expression profiles for identifying the severity of cervical pathological lesions in exfoliated cervical cell samples from HR-HPV-infected women. Six differentially expressed miRNAs were analyzed, including four up-regulated miRNAs (miR-15a-5p, miR-16-5p, miR-20b-5p, and miR-155-5p) and two down-regulated miRNAs (miR-34a-5p and miR-140-3p). MiRNA expression was assessed across five diagnostic categories (Control, NILM, CIN II, CIN III, and cervical cancer [CC]) in a cohort of 100 patients. Additionally, 15 cervical cancer biopsies (TB) were analyzed to compare miRNA expression between exfoliated cytological samples and cervical cancer tissue. All investigated miRNAs were consistently detectable in all samples. Relative expression changes were quantified using fold change (FC), defined as the ratio of median expression in each diagnostic category to the control median, and visualized on a log_2_FC scale (Figure 1E,F). Statistical significance of expression differences relative to controls was assessed using Welch’s *t*-test.

Distinct stage-dependent miRNA expression patterns were observed across the NILM–CIN II–CIN III–CC continuum. The up-regulated miRNAs (miR-15a-5p, miR-16-5p, miR-20b-5p, and miR-155-5p) showed a gradual increase in expression compared with controls, detectable from CIN II, further enhanced in CIN III, and reaching the highest levels in exfoliated CC samples and tumor biopsies (Figure 1A–D). For each up-regulated miRNA, CC and TB samples displayed markedly elevated log_2_FC values, corresponding to expression increases exceeding 10–25-fold relative to controls (Supplemental Table S3). Highly significant up-regulation was observed in CIN III, CC, and TB categories (*p* < 1 × 10^−4^), consistent with activation of proliferative and inflammatory oncogenic miRNA programs.

In contrast, miR-34a-5p and miR-140-3p exhibited a consistent reduction in expression in dysplastic and malignant samples (Figure 1E,F). Down-regulation was evident from CIN II and became more pronounced in CIN III and cervical cancer, both in exfoliated cells and tumor biopsies. In CC and TB samples, expression levels were significantly reduced (log_2_FC < −1 to −2; *p* < 1 × 10^−3^ to *p* < 1 × 10^−4^), in line with progressive loss of tumor-suppressive miRNA activity. These results demonstrate that the pattern of miRNA dysregulation accompanying cervical disease progression from NILM to cervical cancer, supporting the potential value of the analyzed miRNAs as biomarkers of disease severity.

### 3.2. ROC Analysis—Diagnostic Performance of miRNAs

**Figure 2:**
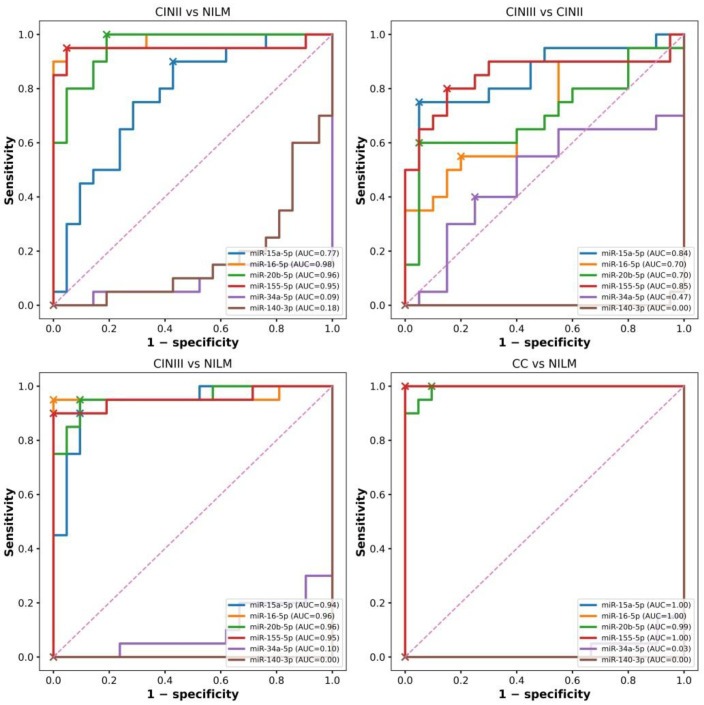
ROC analysis of miRNA expression across progression of cervical lesions. ROC curves for cytology samples comparing CIN II vs. NILM, CIN III vs. CIN II, CIN III vs. NILM, and CC vs. NILM. Crosses (✕) indicate optimal cut-off values determined using the Youden index. The dashed line indicates random prediction (AUC = 0.5).

**Figure 3:**
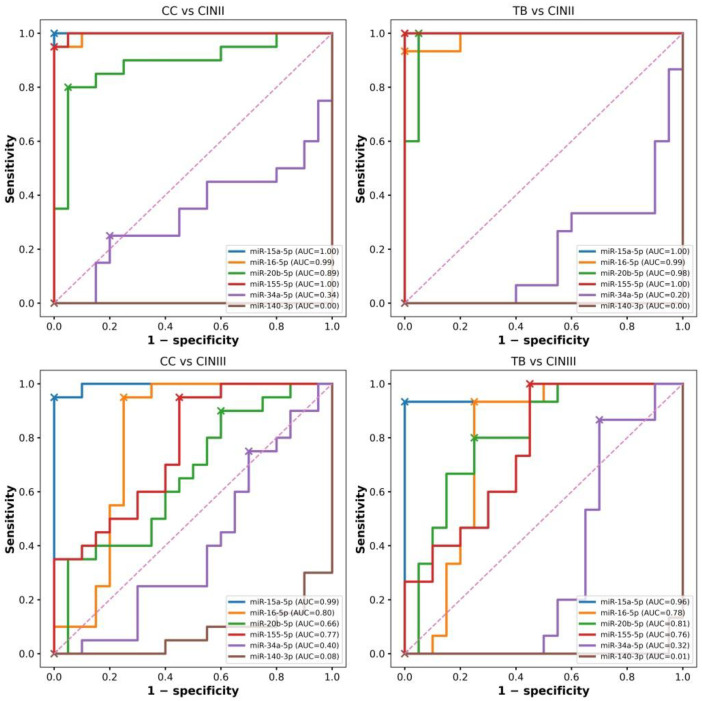
ROC analysis of miRNA expression in cervical cancer. Comparison of miRNA expression in cervical cancer versus CIN II and CIN III lesions analyzed in cervical exfoliated cells (CC) and tissue biopsies (TB). Sensitivity is plotted against 1 − specificity. Crosshairs (✕) indicate optimal cutoff values determined using the Youden index. The dashed line indicates random prediction (AUC = 0.5).

Receiver operating characteristic (ROC) analysis was performed to assess the diagnostic utility of the selected miRNAs in clinically relevant stages of cervical disease progression, including precancerous lesions (CIN II, CIN III) and cervical cancer assessed by cervical exfoliation (CC) and tissue biopsy (TB) (Figure 2 and Figure 3, Supplemental Table S4).

Given the pilot-scale design, all ROC parameters are reported together with corresponding 95% confidence intervals (CIs) to reflect statistical uncertainty. Complete diagnostic metrics, including sensitivity and specificity with 95% CIs at Youden-derived cut-offs, are provided in Supplementary Table S4.

In the comparison of CIN II versus NILM, miR-16-5p demonstrated the highest diagnostic accuracy (AUC = 0.98, 95% CI: 0.94–1.00), followed by miR-20b-5p (AUC = 0.96, 95% CI: 0.89–1.00) and miR-155-5p (AUC = 0.95, 95% CI: 0.87–1.00). In contrast, miR-15a-5p showed more moderate discriminatory performance (AUC = 0.77, 95% CI: 0.62–0.92), indicating reduced precision for distinguishing early dysplastic lesions.

For CIN III versus NILM, all analyzed miRNAs exhibited consistently high discriminatory power, with AUC values ranging from 0.94 to 0.96 (for example, miR-15a-5p: AUC = 0.94 (95% CI: 0.87–1.00)), supporting their potential utility in detecting high-grade lesions (HSIL). Sensitivity and specificity estimates remained in the range of approximately 90–95%, with uncertainty intervals reported in Supplementary Table S4. In the comparison of CC versus NILM, several markers achieved near-perfect diagnostic performance. miR-15a-5p, miR-16-5p, and miR-155-5p, each reached AUC values close to 1.00, for example miR-16-5p: AUC = 1.00 (95% CI: 1.00–1.00), suggesting strong discrimination of invasive cancer in exfoliated cytology specimens.

However, given the limited cohort size, these results should be interpreted cautiously as exploratory estimates requiring validation in independent populations.

Greater variability was observed when distinguishing CIN II from CIN III, reflecting the clinical difficulty of separating adjacent precancer stages. The highest accuracy was observed for miR-155-5p (AUC = 0.85, 95% CI: 0.72–0.98), followed by miR-15a-5p (AUC = 0.84, 95% CI: 0.71–0.97), while miR-16-5p and miR-20b-5p showed only moderate performance (AUC ≈ 0.70). The down-regulated miRNAs miR-34a-5p and miR-140-3p did not show clinically meaningful discriminatory value across the evaluated comparisons (see Supplemental Table S4).

To determine whether miRNA expression profiles would differ depending on the source of the material, miRNA profiles were additionally analyzed in cervical exfoliated cells (CC) and cervical tissue (TB) samples obtained from patients with cervical cancer (Figure 3).

Comparison of cytology (CC) and tissue (TB) samples demonstrated a clear differentiation of cancer from CIN II by miR-155-5p and miR-15a-5p (AUC ≈ 1.00). The discriminatory power of miR-16-5p was slightly lower (AUC = 0.99, 95% CI: 0.96–1.00) and the same in both cytology and biopsy samples. For miR-20b-5p, the AUC was lower in cytology compared to tissue samples (CC: AUC = 0.89 vs. TB: AUC = 0.98), suggesting potential specimen-dependent variability.

Greater variability in miRNA discriminatory power was observed when comparing cervical cancer samples (cytology and tissue) with CINIII. Overall, the discriminatory power of ROC analysis decreased for all miRNAs analyzed (Figure 3 and Supplemental Table S4).

To confirm that the discriminatory levels of the studied miRNAs did not change regardless of whether they were analyzed in exfoliated cervical cells or in cervical tissue, the AUC and Youden’s indices of the analyzed miRNAs were compared between exfoliated cervical cells and tissue biopsies. Pairwise analysis was performed for the same reference category: CC vs. CIN II with TB vs. CIN II and for CC vs. CIN III with TB vs. CIN III, calculating ΔAUC = AUC(TB) − AUC(CC) and ΔYouden = Youden(TB) − Youden(CC). Positive difference values indicated better diagnostic performance in tissue specimens (TB), while values close to 0 indicated high translation of the expression signal to cytology (CC).

miR-15a-5p and miR-16-5p, demonstrated comparable diagnostic performance (|ΔYouden| < 0.05; Supplemental Table S5), confirming their high diagnostic stability as minimally invasive cytology-based biomarkers.

The observed heterogeneous diagnostic performance of individual miRNAs and the variable agreement between cytology and tissue measurements indicate that cervical lesion progression is not driven by uniform changes in single markers corresponding to disease progression, justifying subsequent correlation- and network-based analyses.

### 3.3. Correlation Structure and miRNA Expression Heterogeneity

Initial analysis of relative miRNA expression (log_2_FC) revealed disease progression-dependent differences. On the other hand, the variable diagnostic performance observed in subsequent ROC analyses (CINIII vs. CIN II, Figure 2; CC vs. CINIII, Figure 3) indicated that changes in expression levels alone cannot fully explain cervical lesion progression. The discordance between effect size (log_2_FC) and diagnostic metrics (AUC and Youden index) provided the rationale for further investigation of miRNA co-expression patterns and correlation structure.

**Figure 4:**
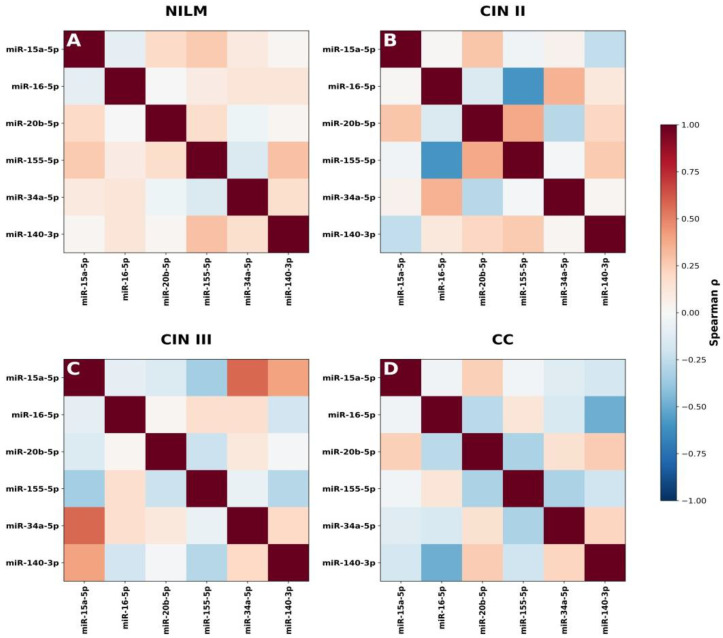
(**A**–**D**) Spearman correlation heatmaps illustrating miRNA co-expression structures within individual diagnostic categories (NILM, CIN II, CIN III, and CC). The diverging color scale reflects the direction and magnitude of correlation coefficients (ρ), depicting the static correlation architecture characteristic of each disease stage. Complete Spearman correlation matrices with numeric data (ρ) for all analyzed miRNAs within each diagnostic category are in Supplemental Table S6.

Correlation analyses demonstrated pronounced differences in miRNA relationships across diagnostic categories. Spearman rank correlation heatmaps revealed that each group (NILM, CIN II, CIN III, and CC) exhibited a distinct, category-specific correlation structure (Figure 4).

These findings indicate increasing regulatory heterogeneity with advancing disease severity and support the hypothesis that cervical disease progression is associated with coordinated changes in miRNA interactions rather than uniform shifts in the expression of individual markers.

### 3.4. Differential Correlation Analysis (Δρ) and Network Remodeling

**Figure 5:**
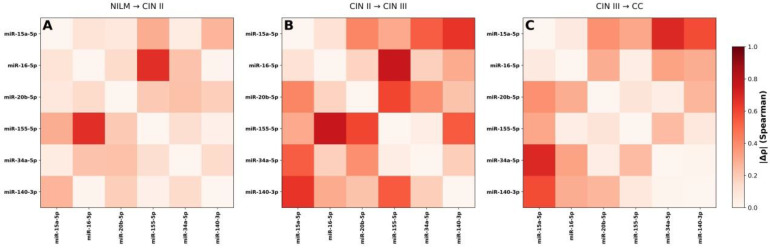
(**A**–**C**) Differential dynamics of miRNA correlations across progression of cervical lesions. Heatmaps show the absolute magnitude of changes in pairwise Spearman correlations (|Δρ|) between consecutive diagnostic categories (NILM to CIN II, CIN II to CIN III, and CIN III to CC). Differential correlation heatmaps with numeric Δρ values are in Supplemental Figure S2.

To quantitatively assess changes in miRNA associations between successive stages of disease progression, differential correlation analysis (Δρ) was performed (Figure 5A–C). Heatmaps depicting the absolute magnitude of changes in Spearman correlation coefficients (|Δρ|) between consecutive diagnostic categories (NILM to CIN II, CIN II to CIN III, and CIN III to CC) revealed a progressive reorganization of miRNA co-expression architecture.

The presence of both positive and negative Δρ values indicates non-uniform rewiring of the regulatory network, encompassing strengthening, weakening, or inversion of pairwise miRNA correlations (Figure 5A–C).

The transition from NILM to CIN II was characterized by early rewiring of miRNA co-expression involving miR-155-5p with miR-16-5p, miR-15a-5p, miR-20b-5p, and miR140-3p, suggesting initial disruption of cell cycle regulatory control. The CIN II to CIN III transition represented a critical inflection point, marked by a pronounced increase in correlation changes, particularly among miR-15a-5p, miR-20b-5p, miR-34a-5p, and miR-155-5p. These changes are consistent with the loss of cell cycle checkpoints and the activation of oncogenic pathways, indicating a significant shift in the regulatory architecture associated with progression risk. In cervical cancer (CC), the newly established correlations persisted, suggesting stabilization of an aggressive regulatory network (Supplemental Figure S2).

### 3.5. Integration of Diagnostic and Network-Level Analyses

**Figure 6:**
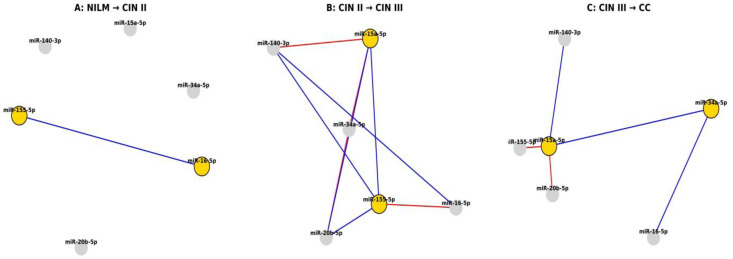
(**A**–**C**) Differential correlation networks (|Δρ| ≥ 0.3) across cervical lesions. The figure illustrates gains and losses of miRNA interactions between consecutive diagnostic categories (NILM to CIN II, CIN II to CIN III, and CIN III to CC). Network hubs (upper quartile of degree distribution) are highlighted in gold, stable miRNAs in gray; red edges indicate strengthening of correlations (Δρ > 0.3), whereas blue edges indicate loss or inversion of correlations (Δρ < −0.3).

Differential correlation network analysis (|Δρ| ≥ 0.3) demonstrated both gains and losses of miRNA interactions during disease progression (Figure 6A–C).

The presented analyses constitute an integrated marker–network approach that combines static measures of diagnostic performance for individual miRNAs (AUC and Youden index) with dynamic measures of their involvement in regulatory network remodeling (mean |Δρ| and network degree) (Supplemental Table S6). This integrated framework enables discrimination between miRNAs with high diagnostic stability and those actively participating in the rewiring of the regulatory network during disease progression.

Integrative analysis revealed that miRNAs with high diagnostic performance do not uniformly correspond to network hubs. In particular, miR-15a-5p and miR-155-5p combined high AUC and Youden index values with substantial involvement in network remodeling, consistent with hub-like behavior. In contrast, miR-16-5p and miR-20b-5p, despite good diagnostic performance, exhibited more stable network relationships. These findings highlight distinct roles of miRNAs as diagnostic biomarkers versus drivers of molecular network dynamics in cervical cancer progression (Supplemental Table S7).

Our results suggest that cervical lesion progression is driven not solely by changes in individual miRNA expression levels but by stage-specific reorganization of the miRNA regulatory network, in which only a subset of diagnostically informative miRNAs actively contribute to network rewiring.

## 4. Discussion

Cervical carcinogenesis is an example of virus-driven cancer, in which persistent infection with high-risk human papillomavirus (HR-HPV) types induces a gradual and multistep remodeling of host–cell regulatory networks. This process involves functional inactivation of the p53 and Rb pathways by the viral oncoproteins E6 and E7, increasing genomic instability, modulation of immune responses, and—at later stages—HPV genome integration, which stabilizes the malignant phenotype [2].

MicroRNAs (miRNAs) participate in these processes and can act as regulatory hubs or messengers that integrate viral, inflammatory, and proliferative signaling. Changes in miRNA expression are not merely secondary epithelial phenomena but actively contribute to transcriptional reprogramming in HPV-infected epithelial cells [26]. Consequently, miRNAs represent both potential diagnostic biomarkers and functional indicators of the molecular state of the cervical epithelium [10,11,40].

Cervical cancer prevention relies on effective screening strategies capable of accurately identifying women at risk of progression while minimizing unnecessary diagnostic procedures. Although HPV DNA testing has substantially improved sensitivity, its limited specificity results in high rates of transient HPV positivity, leading to over-referral for colposcopy and overtreatment. Therefore, clinically applicable biomarkers that improve risk stratification and triage remain a critical unmet need [3,41,42].

In the present study, we focused on six miRNAs with established roles in cell cycle regulation, immune signaling, and genomic stability. The source material consisted of liquid cytology samples. Analysis of relative miRNA expression (log_2_FC) revealed clear, stage-dependent trends from NILM through CIN II and CIN III to invasive cervical cancer, despite the heterogeneity of LBC samples. These observations indicate that miRNA-derived molecular signals are sufficiently robust to be detected in heterogeneous cytological material, underscoring the biological relevance of the selected miRNAs.

For the upregulated miRNAs (miR-15a-5p, miR-16-5p, miR-20b-5p, and miR-155-5p), a gradual increase in expression was observed compared to the control group across a continuum of diagnostic categories (NILM, CINII, CINIII, and CC). Downregulated miR-34a-5p and miR-140-3p showed a consistent reduction in expression in dysplastic and CC samples. The miRNA expression profiles obtained in this study are consistent with previously reported observations obtained in studies of fresh and frozen tissues, which indicate that miR-15a-5p [27,36], miR-16-5p [35], miR-20b-5p [32,43], and miR-155-5p [44,45] demonstrate highly elevated expression levels in lesion tissues compared to controls, i.e., normal cytology and a negative HPV test. Similarly, miR-34a-5p [14,23,24,25,46] and miR-140-3p [37,47,48] are downregulated in malignant tissues.

ROC analyses highlighted substantial heterogeneity in diagnostic performance among individual miRNAs. Downregulated miRNAs, including miR-34a-5p and miR-140-3p, showed limited diagnostic value, suggesting that gradual loss of cancer-suppressive signals does not generate clinically actionable thresholds in cytology-based samples.

Recent studies have demonstrated that miRNA-140-3p suppresses malignant tumor growth. It inhibits cell proliferation and metastasis in cervical cancer [37] and breast cancer [47]. In vivo studies indicated low expression of this anti-oncomiR [48]. Our study analyzed miR-140-3p expression in the continuum of cervical carcinogenesis—virus-induced cancer—and in heterogeneous liquid-based cytology samples. These two factors may have underpinned the insufficient expression variation and, therefore, the limited diagnostic value of miR140-3p.

In turn, reduced miR-34a expression is attributed to the expression of the HR-HPV E6 oncoprotein, which destabilizes the cancer suppressor p53, a known transactivator of miR-34a [14,34,49]. It has also been observed that the productive phase of HR-HPV, associated with the expression of oncoprotein E6, leads to reduced miR34a-5p expression already in precancerous lesions [25]. In our study, it is likely that the presence of the virus itself, starting from NILM, led to decreased miR-34a-5p expression along the NILM-CINII-CINIII-CC continuum, which accounted for the low diagnostic value in the ROC analysis. This result indicated that down-regulated miRNAs are less useful for screening and patient stratification, particularly when liquid-based cytology is used as the starting material.

In contrast, upregulated miRNAs showed substantially higher diagnostic performance, particularly in comparisons between distant clinical categories. The high accuracy regarding the AUC values observed for miR-16-5p, miR-20b-5p, and miR-155-5p and the high-to-moderate accuracy of the AUC for miR-15a-5p are consistent with their established roles as active mediators of proliferation [36,41,43,50].

Importantly, differentiation between adjacent disease stages, such as CIN II and CIN III, as well as CC and CIN III, remained difficult, even for miRNAs with high overall AUC values. This finding has direct clinical relevance because it reflects the biological continuum of cervical carcinogenesis and highlights the limitations of relying on single biomarkers for precise lesion stratification.

To address these limitations, we used correlation and network analyses to capture coordinated changes in miRNA regulation. These analyses provide insight into disease biology rather than direct diagnostic readouts. Correlation and network differentiation analyses demonstrated that the miRNA regulatory architecture undergoes gradual reorganization during disease progression. In NILM samples, miRNAs exhibit weak, diffuse correlations, consistent with physiological regulatory independence. The transition to CIN II is accompanied by the emergence of coordinated interactions between key cell cycle regulators. CIN III represents a critical inflection point characterized by a pronounced densification of the miRNA correlation network, reflecting the convergence of proliferative pathways. In cervical cancer, the newly established correlations persist and stabilize, suggesting the consolidation of a regulatory architecture consistent with HPV genome integration, chromatin remodeling, and microenvironmental modulation.

Taken together, these findings indicate that miRNAs in LBC samples should not be viewed solely as individual diagnostic markers. Their greatest biological and clinical value is revealed at the network level, where miRNA interactions capture key transition points in cervical carcinogenesis—from episomal HPV infection to viral integration and from potentially reversible dysplasia to irreversible neoplastic transformation.

Importantly, miRNAs with strong diagnostic performance did not uniformly correspond to network hubs. For example, miR-15a-5p and miR-155-5p combined good diagnostic accuracy with active participation in network remodeling, whereas miR-16-5p and miR-20b-5p showed high diagnostic performance but greater network stability. This distinction suggests that some miRNAs may function primarily as robust clinical markers, while others reflect underlying regulatory instability associated with progression risk.

From a translational standpoint, these findings support a dual interpretation of miRNA biomarkers—(i) stable, high-performing miRNAs are best suited for diagnostic triage, whereas (ii) miRNAs involved in network rewiring may serve as indicators of biological aggressiveness and progression potential, potentially informing longitudinal risk assessment rather than single-time-point diagnosis.

This study, however, has some limitations that make it a preliminary approach to understanding miRNA regulatory systems in carcinogenesis. The analysis was performed on a predefined panel of six miRNAs, which, although biologically and clinically relevant, does not capture the full complexity of the miRNA regulatory landscape. Broader profiling may identify additional markers or combinations with improved diagnostic performance [12,23,26,28]. Although several miRNAs achieved very high AUC values, these estimates were derived from a pilot-scale case–control design with relatively small group sizes. Because cut-off thresholds were selected using the same dataset, diagnostic performance may be overestimated. Therefore, these findings should be interpreted as exploratory and hypothesis-generating. Larger prospective studies with independent validation cohorts and cross-validation frameworks will be essential to confirm robustness and clinical utility.

Furthermore, longitudinal studies are necessary to determine whether miRNA remodeling at the network level predicts actual progression from CIN II to CIN III or regression to NILM [51]. Although liquid-based cytology provides accurate screening material, its inherent heterogeneity may attenuate expression differences and reduce the power to differentiate subtle stage differences [42,52]. This limitation likely contributed to miRNAs’ reduced ability to distinguish between adjacent precancerous stages in our study. To increase diagnostic specificity, both modifications to molecular tests are necessary, and greater use of informatics/statistical tools is becoming essential [53].

From a biological perspective, our studies indicate that progression from CIN to CC results from coordinated deregulation of miRNA regulatory networks rather than a set of independent expression changes. Restructuring of miRNA network architecture reflects reprogramming of key pathways of neoplastic transformation [9,12,26,35,41,51]. The most promising miRNAs for clinical applications are those that combine high diagnostic efficiency with network stability. Integrating ROC-based diagnostics with network-level analyses enables distinguishing clinically useful biomarkers from miRNAs primarily involved in regulatory rewiring, thereby improving risk stratification for CC.

When considering the potential implementation of miRNA profiling in the diagnosis of HPV-positive women, it is worth emphasizing that HPV genotyping alone allows for differentiation of disease risk (HR-HPV vs. LR-HPV). Still, it does not determine the current stage of disease progression (e.g., HPV16 may be present in NILM and CC), nor does it predict progression (most HPV infections are transient). MiRNA profiling offers the potential to provide a true biological picture.

From a cost-effectiveness perspective, HPV DNA genotyping offers excellent sensitivity but is limited by low specificity, leading to increased referrals for colposcopy and higher treatment costs. In this context, miRNA-based expression profiling may represent a valuable, economically valuable complementary tool, as improved molecular stratification of HPV-positive women may reduce unnecessary diagnostic interventions while focusing resources on patients at high risk of progression.

Currently, RT-qPCR platforms are widely available in diagnostic laboratories, and miRNA assays can be standardized like other molecular screening tests, increasing the likelihood of clinical validation. However, successful implementation across multiple centers will require harmonized sample-processing protocols, standardized strategies, and interlaboratory quality control schemes. Therefore, large-scale, prospective validation studies will be crucial to ensure reproducibility and the regulatory role of specific miRNA expression as biomarkers in routine cervical cancer screening.

## 5. Conclusions

Changes in miRNA expression alone are insufficient to explain cervical disease progression. Although differences in expression related to disease stage are evident, their magnitude does not directly translate into diagnostic efficacy or regulatory significance.

The diagnostic value of miRNAs is heterogeneous and dependent on disease stage. Only a subset/set of miRNAs offers the potential to reveal stable differentiation across diagnostic categories, highlighting the limitations of single-marker approaches.

The progression of cervical lesions is associated with remodeling of miRNA regulatory networks. Differential correlation analyses reveal increased remodeling of miRNA interactions across subsequent disease stages, reflecting systemic deregulation.

MiRNAs play distinct roles as diagnostic biomarkers and network regulators. MiRNAs with high ROC efficiency do not necessarily function as network hubs, and miRNAs that drive network remodeling are not always optimal diagnostic markers.

Progression of cervical lesions is driven not only by changes in miRNA expression levels but also by stage-specific reprogramming of miRNA regulatory networks. Combining diagnostic efficiency with differential correlation analysis allows us to distinguish stable clinical biomarkers from miRNAs that drive regulatory remodeling during carcinogenesis.

As this work represents a pilot exploratory analysis, these findings should be interpreted with caution and validated in larger, independent prospective cohorts prior to potential translation into routine HPV-positive triage workflows.

## 6. Future Perspectives

The studies presented here, using a miRNA panel including miR-15a-5p, miR-155-5p, miR-16-5p, and miR-20b-5p, highlight their potential for distinguishing NILM from high-grade lesions and cervical cancer. However, these results require validation and rigorous analysis in larger multicenter cohorts to confirm their usefulness in triaging HPV-positive patients. Because the regulatory interactions between miRNAs are not fully understood, long-term follow-up studies are necessary, particularly in women with CIN II, to determine whether miRNA profiles and patterns of network remodeling can predict actual progression to CIN III or regression to low-risk states. Furthermore, miRNA profiling methods, such as high-throughput sequencing, may identify additional markers or marker combinations that improve differentiation between adjacent disease stages, a major clinical challenge. Expanded miRNA panels could increase specificity while retaining the minimally invasive advantages of liquid-based cytology sampling.

In turn, integrating miRNA biomarkers with established screening methods—such as HPV genotyping and cytology results, in conjunction with currently recognized methylation-based markers [54]—may enable the development of multiparametric risk-stratification algorithms that are more effective than single-marker strategies and reduce unnecessary colposcopy referrals.

Finally, the network-level analyses framework presented here offers a promising avenue for revealing regulatory instability during cervical carcinogenesis. Future work should investigate whether differential correlation indices (Δρ) and node dynamics may serve not only as descriptive tools but also as predictors of biological aggressiveness. Expanding this approach with additional epigenetic and transcriptomic data may provide deeper insights into HPV-induced transformation and support more precise, personalized screening strategies.

## Data Availability

The datasets generated or analyzed in this study can be obtained from the corresponding author upon reasonable request.

## Supplementary Materials

The following supporting information can be downloaded at: https://www.mdpi.com/article/10.3390/cancers18040559/s1. Figure S1: Workflow of specimen collection and miRNA triage using residual LBC material; Figure S2: Differential correlation heatmaps display signed differences in Spearman correlation coefficients (Δρ) between diagnostic categories, with numeric values; Table S1: General characteristics of the tested patient samples in relation to diagnostic categories; Table S2: List of primer sequences used in RT-qPCR; Table S3: Fold change of miRNA expression in specific diagnostic categories and Welch’s *t*-test *p*-value; Table S4: ROC curves parameters for miRNAs in the analyzed comparisons of diagnostic categories; Table S5: Comparison of the discriminatory power of the studied miRNAs in exfoliated cells and biopsy tissue; Table S6: Complete Spearman correlation matrices (ρ) for all analyzed miRNAs within each diagnostic category (NILM, CIN II, CIN III, and CC); Table S7: Integration of diagnostic efficacy and miRNAs network remodeling in cervical lesion progression.
