# Supplementary material for: MicroRNA-Based Triage of HPV-Positive Women Using Liquid-Based Cytology: Diagnostic Performance and Network-Level Insights
Source: Cancers (Basel). 2026 Feb 9;18(4):559. doi: 10.3390/cancers18040559 (PMC12938343; doi:10.3390/cancers18040559)
Supplement: Supplementary file 1 [file cancers-18-00559-s001.zip › cancers-4112291-supplementary.pdf]

Supplementary materials

MicroRNA-Based Triage of HPV-Positive Women Using Liquid-Based Cytology: Diagnostic Performance and Network-Level Insights

Justyna Pisarska[1], Aleksandra Kożańska[1], Rafał Rzepka[2] and Katarzyna Baldy-Chudzik[1]*

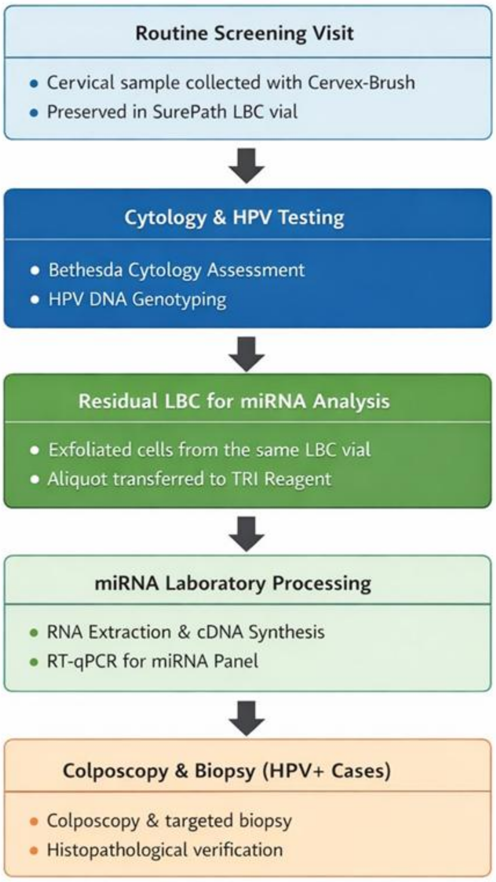

Figure S1. Workflow of specimen collection and miRNA triage using residual LBC material.

All miRNA analyses were performed using residual exfoliated cervical cells derived from the same SurePath LBC specimen collected during the routine HPV screening visit, without requiring additional sampling prior to colposcopy.

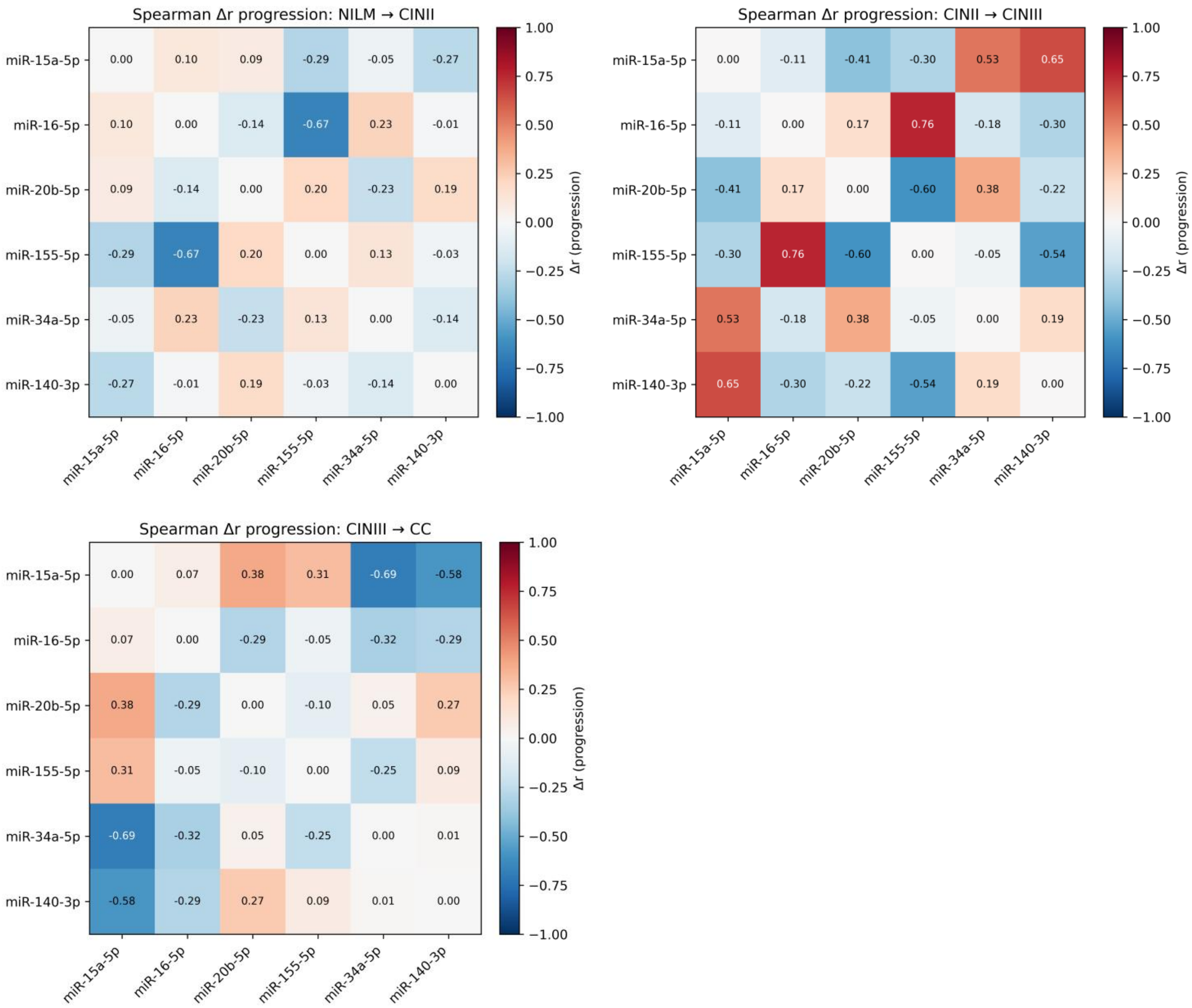

Figure S2. Differential miRNA correlation heatmaps, with numeric Δρ values, across cervical lesion progression.

Heatmaps display pairwise differences in Spearman correlation coefficients (Δρ) between consecutive diagnostic categories (CIN II vs NILM, CIN III vs CIN II, and CC vs CIN III). Positive Δρ values (red) indicate strengthening of miRNA co-expression, whereas negative values (blue) denote loss or inversion of correlations during progression. Numeric values within heatmap cells represent Δρ for individual miRNA pairs, highlighting stage-specific remodeling of miRNA interaction patterns beyond changes in expression levels.

Table S1. General characteristics of the tested patient samples in relation to diagnostic categories

Verified by colposcopy	Healthy control HPV-	CIN 0	CINII	CINIII	CC	Tissue Biopsy (TB)
Number in group	20	20	20	20	20	15
Age (median)	34	41	38	39	31	31
Cytology prior to colposcopy:						
NILM	20(100%)	20(100%)	0	0	0	0
ASC-US	-	0	1 (5%)	0	0	0
LSIL	-	0	8 (40%)	1 (5%)	0	0
HSIL	-	0	11(55%)	18(90%)	1(5%)	0
CC	-	0	0	1 (5%)	19(95%)	15(100%)
HR-HPV positivity:						
HPV16	0	6	8	10	16	12
HPV16+HPV31	0	3	3	7	2	2
HPV16+HPV18+HPV33	0	2	0	0	1	1
HPV!6+HPV53	0	4	4	0	1	0
HPV16+HPV56+HPV53	0	1	2	0	0	0
HPV16+HPV6	0	4	3	3	0	0

This table characterizes the tested cervical smear and biopsy material, taking into account the cytological and histological diagnosis and HPV genotypes in single and multiple infections.

Table S2. Primer sequences used in RT-qPCR

**Target**	**Primer sequences (5' – 3')**
miR-34a-5p	F: TGGCAGTGTCTTAGCTGGTTGT
miR-15a-5p	F: TAGCAGCACATAATGGTTTGTG
miR-16-5p	F:TAGCAGCACGTAAATATTGGCG
miR-20b-5p	F:CAAAGTGCTCATAGTGCAGGTAG
miR-155-5p	F: TTAATGCTAATCGTGATAGGGGT
miR-140-3p	F: TAGGGCTACCACAGGGTAGAA
miR-423	F: AGCTCGGTCTGAGGCCCCTCAGT
Rnu43	F: AGAGCCAAATCAGAACGTGA<br>R: CTCGCAGAAACCACAGATGAT

The table contains the sequences of F primers for the analyzed miRNAs and the reference genes (miR-423 and Rnu43) used as endogenous controls in RT-qPCR reactions for expression analysis and their normalization.

Table S3. Fold change of miRNA expression in specific diagnostic categories and Welch's t-test p-value.

The table summarizes the expression values of the studied miRNAs across individual diagnostic categories, with median ± SEM (Standard Error of the Mean), Fold Change, and statistical significance (p) in Welch's t-test compared to the control.

**miRNA**	**Category**	**Median**	**SEM**	**FC_vs_Control**	**p_Welch**
miR-15a-5p	Control	0,1872287	0,20069264		
miR-15a-5p	NILM	0,5341343	0,101232037	2,852844142	0,555593248
miR-15a-5p	CINII	1,00577417	0,087268592	5,37190169	0,034030432
miR-15a-5p	CINIII	2,0357863	0,12076959	10,87325982	1,06214E-05
miR-15a-5p	CC	3,6898085	0,168341386	19,7074941	2,03424E-14
miR-15a-5p	TB	3,9768564	0,219923566	21,24063458	1,13881E-12
miR-16-5p	Control	0,381857	0,059157986		
miR-16-5p	NILM	0,5238438	0,09428724	1,371832388	0,156042299
miR-16-5p	CINII	3,77412915	0,429843126	9,88361913	9,40519E-08
miR-16-5p	CINIII	5,6188602	0,770283904	14,71456645	4,70878E-07
miR-16-5p	CC	10,25945005	0,282494658	26,86725672	2,24949E-19
miR-16-5p	TB	9,7653244	0,393064293	25,57324967	5,21205E-13
miR-20b-5p	Control	0,49868495	0,136356904		
miR-20b-5p	NILM	0,4108273	0,119843704	0,823821332	0,771839194
miR-20b-5p	CINII	2,668561	0,36956901	5,351196181	4,89092E-06
miR-20b-5p	CINIII	5,6197153	0,616081832	11,26906938	1,01558E-06
miR-20b-5p	CC	6,44949605	0,530310812	12,9330072	4,01527E-10
miR-20b-5p	TB	7,8475634	0,340777639	15,73651541	1,20662E-13
miR-155-5p	Control	0,6215935	0,087958397		
miR-155-5p	NILM	0,6269005	0,113423149	1,008537734	0,743278031
miR-155-5p	CINII	4,05663255	0,432983939	6,526182384	3,07853E-07
miR-155-5p	CINIII	8,5699472	0,883063451	13,78706051	2,92242E-08
miR-155-5p	CC	12,1532949	0,748447338	19,55183717	1,25981E-12
miR-155-5p	TB	11,7359223	0,54082912	18,88038131	1,94602E-12
miR-34a-5p	Control	1,37517125	0,148590258		
miR-34a-5p	NILM	1,4748813	0,142184023	1,07250737	0,156993388
miR-34a-5p	CINII	0,3291891	0,117108781	0,239380441	0,000221908
miR-34a-5p	CINIII	0,3635276	0,119351126	0,264350785	0,000414654
miR-34a-5p	CC	0,2067035	0,068375736	0,150311098	4,60229E-06
miR-34a-5p	TB	0,1895643	0,020226938	0,137847777	7,11989E-07
miR-140-3p	Control	1,55842015	0,089722423		
miR-140-3p	NILM	1,3267485	0,098753006	0,851341982	0,578294847
miR-140-3p	CINII	0,99771605	0,051411455	0,640209927	0,000161622
miR-140-3p	CINIII	0,50559055	0,039034238	0,32442506	2,96731E-10
miR-140-3p	CC	0,27464445	0,028190727	0,17623261	3,233E-12
miR-140-3p	TB	0,1934765	0,016073279	0,124149126	3,87603E-12

Table S4. ROC curves parameters for miRNAs in the analyzed comparisons of diagnostic categories.

Comparison	Marker	AUC (95% CI)	Cut-off	Youden (J)	Sensitivity (95% CI)	Specificity (95%CI)	PPV	NPV	N
**CINII vs NILM**	miR-15a-5p	0.77 (0.62–0.92)	0.58	0.47	0.90 (0.77–0.96)	0.57 (0.42–0.71)	0.67	0.86	41
**CINII vs NILM**	miR-16-5p	0.98 (0.94–1.00)	1.50	0.90	0.95 (0.84–0.99)	0.95 (0.84–0.99)	0.95	0.95	41
**CINII vs NILM**	miR-20b-5p	0.96 (0.89–1.00)	0.79	0.81	1.00 (0.91–1.00)	0.81 (0.67–0.90)	0.83	1.00	41
**CINII vs NILM**	miR-155-5p	0.95 (0.84–1.00)	1.48	0.90	0.95 (0.84–0.99)	0.95 (0.84–0.99)	0.95	0.95	41
**CINII vs NILM**	miR-34a-5p	0.09 (0.00–0.19)	inf	0.00	0.00 (0.00–0.09)	1.00 (0.91–1.00)		0.51	41
**CINII vs NILM**	miR-140-3p	0.18 (0.07–0.32)	inf	0.00	0.00 (0.00–0.09)	1.00 (0.91–1.00)		0.51	41
**CINIII vs NILM**	miR-15a-5p	0.94 (0.85–1.00)	1.11	0.80	0.90 (0.77–0.96)	0.90 (0.78–0.96)	0.90	0.90	41
**CINIII vs NILM**	miR-16-5p	0.96 (0.87–1.00)	2.21	0.95	0.95 (0.84–0.99)	1.00 (0.91–1.00)	1.00	0.95	41
**CINIII vs NILM**	miR-20b-5p	0.96 (0.88–1.00)	1.49	0.85	0.95 (0.84–0.99)	0.90 (0.78–0.96)	0.90	0.95	41
**CINIII vs NILM**	miR-155-5p	0.95 (0.87–1.00)	4.48	0.90	0.90 (0.77–0.96)	1.00 (0.91–1.00)	1.00	0.91	41
**CINIII vs NILM**	miR-34a-5p	0.10 (0.02–0.22)	inf	0.00	0.00 (0.00–0.09)	1.00 (0.91–1.00)		0.51	41
**CINIII vs NILM**	miR-140-3p	0.00 (0.00–0.00)	inf	0.00	0.00 (0.00–0.09)	1.00 (0.91–1.00)		0.51	41
**CC vs NILM**	miR-15a-5p	1.00 (1.00–1.00)	2.25	1.00	1.00 (0.91–1.00)	1.00 (0.91–1.00)	1.00	1.00	41
**CC vs NILM**	miR-16-5p	1.00 (1.00–1.00)	7.35	1.00	1.00 (0.91–1.00)	1.00 (0.91–1.00)	1.00	1.00	41
**CC vs NILM**	miR-20b-5p	0.99 (0.97–1.00)	1.65	0.90	1.00 (0.91–1.00)	0.90 (0.78–0.96)	0.91	1.00	41
**CC vs NILM**	miR-155-5p	1.00 (1.00–1.00)	7.98	1.00	1.00 (0.91–1.00)	1.00 (0.91–1.00)	1.00	1.00	41
**CC vs NILM**	miR-34a-5p	0.03 (0.00–0.08)	inf	0.00	0.00 (0.00–0.09)	1.00 (0.91–1.00)		0.51	41
**CC vs NILM**	miR-140-3p	0.00 (0.00–0.00)	inf	0.00	0.00 (0.00–0.09)	1.00 (0.91–1.00)		0.51	41
**CINIII vs CINII**	miR-15a-5p	0.84 (0.69–0.96)	1.43	0.70	0.75 (0.60–0.86)	0.95 (0.83–0.99)	0.94	0.79	40
**CINIII vs CINII**	miR-16-5p	0.70	5.39	0.35	0.55	0.80	0.73	0.64	40

		(0.51–0.85)			(0.40–0.69)	(0.65–0.90)			
**CINIII vs CINII**	miR-20b-5p	0.70<br>(0.53–0.88)	4.88	0.55	0.60<br>(0.45–0.74)	0.95<br>(0.83–0.99)	0.92	0.70	40
**CINIII vs CINII**	miR-155-5p	0.85<br>(0.71–0.97)	6.02	0.65	0.80<br>(0.65–0.90)	0.85<br>(0.71–0.93)	0.84	0.81	40
**CINIII vs CINII**	miR-34a-5p	0.47<br>(0.28–0.67)	0.41	0.15	0.40<br>(0.26–0.55)	0.75<br>(0.60–0.86)	0.62	0.56	40
**CINIII vs CINII**	miR-140-3p	0.00<br>(0.00–0.02)	inf	0.00	0.00<br>(0.00–0.09)	1.00<br>(0.91–1.00)		0.50	40
**CC vs CINII**	miR-15a-5p	1.00<br>(1.00–1.00)	2.25	1.00	1.00<br>(0.91–1.00)	1.00<br>(0.91–1.00)	1.00	1.00	40
**CC vs CINII**	miR-16-5p	0.99<br>(0.98–1.00)	8.15	0.95	0.95<br>(0.83–0.99)	1.00<br>(0.91–1.00)	1.00	0.95	40
**CC vs CINII**	miR-20b-5p	0.89<br>(0.77–0.98)	4.98	0.75	0.80<br>(0.65–0.90)	0.95<br>(0.83–0.99)	0.94	0.83	40
**CC vs CINII**	miR-155-5p	1.00<br>(0.98–1.00)	9.13	0.95	0.95<br>(0.83–0.99)	1.00<br>(0.91–1.00)	1.00	0.95	40
**CC vs CINII**	miR-34a-5p	0.34<br>(0.16–0.51)	0.44	0.05	0.25<br>(0.14–0.40)	0.80<br>(0.65–0.90)	0.56	0.52	40
**CC vs CINII**	miR-140-3p	0.00<br>(0.00–0.00)	inf	0.00	0.00<br>(0.00–0.09)	1.00<br>(0.91–1.00)		0.50	40
**CC vs CINIII**	miR-15a-5p	0.99<br>(0.98–1.00)	2.82	0.95	0.95<br>(0.83–0.99)	1.00<br>(0.91–1.00)	1.00	0.95	40
**CC vs CINIII**	miR-16-5p	0.80<br>(0.63–0.94)	8.15	0.70	0.95<br>(0.83–0.99)	0.75<br>(0.60–0.86)	0.79	0.94	40
**CC vs CINIII**	miR-20b-5p	0.66<br>(0.47–0.80)	3.98	0.30	0.90<br>(0.77–0.96)	0.40<br>(0.26–0.55)	0.60	0.80	40
**CC vs CINIII**	miR-155-5p	0.77<br>(0.61–0.90)	9.13	0.50	0.95<br>(0.83–0.99)	0.55<br>(0.40–0.69)	0.68	0.92	40
**CC vs CINIII**	miR-34a-5p	0.40<br>(0.23–0.58)	0.17	0.05	0.75<br>(0.60–0.86)	0.30<br>(0.18–0.45)	0.52	0.55	40
**CC vs CINIII**	miR-140-3p	0.08<br>(0.01–0.17)	inf	0.00	0.00<br>(0.00–0.09)	1.00<br>(0.91–1.00)		0.50	40
**TB vs CINII**	miR-15a-5p	1.00<br>(0.98–1.00)	1.86	0.95	1.00<br>(0.90–1.00)	0.95<br>(0.82–0.99)	0.94	1.00	35
**TB vs CINII**	miR-16-5p	0.99<br>(0.95–1.00)	7.98	0.93	0.93<br>(0.80–0.98)	1.00<br>(0.90–1.00)	1.00	0.95	35
**TB vs CINII**	miR-20b-5p	0.98<br>(0.93–1.00)	4.96	0.95	1.00<br>(0.90–1.00)	0.95<br>(0.82–0.99)	0.94	1.00	35
**TB vs CINII**	miR-155-5p	1.00<br>(1.00–1.00)	9.28	1.00	1.00<br>(0.90–1.00)	1.00<br>(0.90–1.00)	1.00	1.00	35
**TB vs CINII**	miR-34a-5p	0.20<br>(0.07–0.36)	inf	0.00	0.00<br>(0.00–0.10)	1.00<br>(0.90–1.00)		0.57	35
**TB vs CINII**	miR-140-3p	0.00	inf	0.00	0.00	1.00		0.57	35

		(0.00–0.00)			(0.00–0.10)	(0.90–1.00)			
**TB vs CINIII**	miR-15a-5p	0.96<br>(0.88–1.00)	2.97	0.93	0.93<br>(0.80–0.98)	1.00<br>(0.90–1.00)	1.00	0.95	35
**TB vs CINIII**	miR-16-5p	0.78<br>(0.59–0.93)	7.98	0.68	0.93<br>(0.80–0.98)	0.75<br>(0.59–0.86)	0.74	0.94	35
**TB vs CINIII**	miR-20b-5p	0.81<br>(0.64–0.94)	6.79	0.55	0.80<br>(0.64–0.90)	0.75<br>(0.59–0.86)	0.71	0.83	35
**TB vs CINIII**	miR-155-5p	0.76<br>(0.58–0.90)	9.28	0.55	1.00<br>(0.90–1.00)	0.55<br>(0.39–0.70)	0.62	1.00	35
**TB vs CINIII**	miR-34a-5p	0.32<br>(0.16–0.54)	0.16	0.17	0.87<br>(0.72–0.94)	0.30<br>(0.17–0.47)	0.48	0.75	35
**TB vs CINIII**	miR-140-3p	0.01<br>(0.00–0.03)	inf	0.00	0.00<br>(0.00–0.10)	1.00<br>(0.90–1.00)		0.57	35

Table S5. Comparison of the discriminatory power of the studied miRNAs in exfoliated cells and biopsy tissue.

The table shows differences in AUC and Youden's coefficient, confirming that the expression levels of the studied miRNAs do not differ between exfoliated cervical cells and cervical tissue.

**Comparison**	**miRNA**	**AUC_CC**	**AUC_TB**	**ΔAUC (TB−CC)**	**Youden_CC**	**Youden_TB**	**ΔYouden (TB−CC)**
CC vs TB (ref: CINII)	miR-15a-5p	1	0,997	-0,003	1	0,95	-0,05
CC vs TB (ref: CINII)	miR-16-5p	0,995	0,987	-0,008	0,95	0,933	-0,017
CC vs TB (ref: CINII)	miR-20b-5p	0,888	0,98	0,092	0,75	0,95	0,2
CC vs TB (ref: CINII)	miR-155-5p	0,997	1	0,003	0,95	1	0,05
CC vs TB (ref: CINII)	miR-34a-5p	0,335	0,197	-0,138	0,05	0	-0,05
CC vs TB (ref: CINII)	miR-140-3p	0	0	0	0	0	0
CC vs TB (ref: CINIII)	miR-15a-5p	0,995	0,963	-0,032	0,95	0,933	-0,017
CC vs TB (ref: CINIII)	miR-16-5p	0,8	0,777	-0,023	0,7	0,683	-0,017
CC vs TB (ref: CINIII)	miR-20b-5p	0,658	0,81	0,152	0,3	0,55	0,25
CC vs TB (ref: CINIII)	miR-155-5p	0,765	0,76	-0,005	0,5	0,55	0,05
CC vs TB (ref: CINIII)	miR-34a-5p	0,4	0,323	-0,077	0,05	0,167	0,117
CC vs TB (ref: CINIII)	miR-140-3p	0,077	0,007	-0,07	0	0	0

The analysis was performed pairwise against the same reference category:

CC vs. CIN II ↔ TB vs. CIN II

CC vs. CIN III ↔ TB vs. CIN III

ΔAUC = AUC(TB) − AUC(CC) ΔYouden = Youden(TB) − Youden(CC)

Positive values = better diagnostic performance in tissue material (TB)

Values close to 0 = high signal translation to cytology (CC)

miRNAs with high translational signal for cytology - based on Table S5.

**Comparison**	**miRNA**	**AUC _CC**	**AUC _TB**	**ΔAUC (TB−CC)**	**Youden _CC**	**Youden _TB**	**ΔYouden (TB−CC)**	**Translational _status**
CC vs TB (ref: CINII)	miR-16-5p	0,995	0,987	-0,008	0,95	0,933	-0,017	Transl. robust (|ΔYouden| < 0.05)
CC vs TB (ref: CINII)	miR-140-3p	0	0	0	0	0	0	Transl. robust (|ΔYouden| < 0.05)
CC vs TB (ref: CINIII)	miR-15a-5p	0,995	0,963	-0,032	0,95	0,933	-0,017	Transl. robust (|ΔYouden| < 0.05)
CC vs TB (ref: CINIII)	miR-16-5p	0,8	0,777	-0,023	0,7	0,683	-0,017	Transl. robust (|ΔYouden| < 0.05)
CC vs TB (ref: CINIII)	miR-140-3p	0,077	0,007	-0,07	0	0	0	Transl. robust (|ΔYouden| < 0.05)

Table S6. Complete Spearman correlation matrices (ρ) for all analyzed miRNAs within each diagnostic category (NILM, CIN II, CIN III, and CC)

Rho (ρ) NILM

	miR-15a-5p	miR-16-5p	miR-20b-5p	miR-155-5p	miR-34a-5p	miR-140-3p
**miR-15a-5p**	1	-0,091	0,188	0,258	0,095	0,023
**miR-16-5p**	-0,091	1	-0,006	0,079	0,122	0,118
**miR-20b-5p**	0,188	-0,006	1	0,178	-0,045	0,023
**miR-155-5p**	0,258	0,079	0,178	1	-0,145	0,294
**miR-34a-5p**	0,095	0,122	-0,045	-0,145	1	0,157
**miR-140-3p**	0,023	0,118	0,023	0,294	0,157	1

Rho CIN II

	miR-15a-5p	miR-16-5p	miR-20b-5p	miR-155-5p	miR-34a-5p	miR-140-3p
**miR-15a-5p**	1	0,012	0,274	-0,033	0,041	-0,245
**miR-16-5p**	0,012	1	-0,144	-0,591	0,348	0,107
**miR-20b-5p**	0,274	-0,144	1	0,38	-0,274	0,211
**miR-155-5p**	-0,033	-0,591	0,38	1	-0,018	0,259
**miR-34a-5p**	0,041	0,348	-0,274	-0,018	1	0,017
**miR-140-3p**	-0,245	0,107	0,211	0,259	0,017	1

Rho CIN III

	miR-15a-5p	miR-16-5p	miR-20b-5p	miR-155-5p	miR-34a-5p	miR-140-3p
**miR-15a-5p**	1	-0,101	-0,137	-0,337	0,573	0,404
**miR-16-5p**	-0,101	1	0,023	0,17	0,171	-0,193
**miR-20b-5p**	-0,137	0,023	1	-0,215	0,107	-0,008
**miR-155-5p**	-0,337	0,17	-0,215	1	-0,063	-0,284
**miR-34a-5p**	0,573	0,171	0,107	-0,063	1	0,202
**miR-140-3p**	0,404	-0,193	-0,008	-0,284	0,202	1

Rho CC

	miR-15a-5p	miR-16-5p	miR-20b-5p	miR-155-5p	miR-34a-5p	miR-140-3p
**miR-15a-5p**	1	-0,032	0,239	-0,026	-0,117	-0,174
**miR-16-5p**	-0,032	1	-0,269	0,122	-0,15	-0,486
**miR-20b-5p**	0,239	-0,269	1	-0,314	0,153	0,257
**miR-155-5p**	-0,026	0,122	-0,314	1	-0,313	-0,198
**miR-34a-5p**	-0,117	-0,15	0,153	-0,313	1	0,215
**miR-140-3p**	-0,174	-0,486	0,257	-0,198	0,215	1

TableS7. Integration of diagnostic efficacy and miRNA network remodeling in cervical lesion progression.

**miRNA**	**AUC_mean**	**Youden_mean**	**|Δρ|mean**	**network_degree**	**network_role**
miR-140-3p	0,033154762	0	0,252	3	stable
miR-155-5p	0,919657738	0,828547619	0,291	4	hub
miR-15a-5p	0,947767857	0,862190476	0,322	4	hub
miR-16-5p	0,91147619	0,823630952	0,246	3	stable
miR-20b-5p	0,888392857	0,748630952	0,246	3	stable
miR-34a-5p	0,243035714	0,052083333	0,228	3	stable

Comparing mean AUC values, Youden index, |Δρ|, and network parameters enables discrimination between miRNAs with high translational stability and those involved in regulatory architecture remodeling.
